# Supplementary material for: Impact of preventive chemotherapy on transmission of soil-transmitted helminth infections in Pemba Island, United Republic of Tanzania, 1994–2021
Source: PLoS Negl Trop Dis. 2022 Jun 27;16(6):e0010477. doi: 10.1371/journal.pntd.0010477 (PMC9236265; doi:10.1371/journal.pntd.0010477)
Supplement: Attachment — Submitted filename: Response to reviewer.docx [file pntd.0010477.s003.docx]

**Response to reviewer**

We would like to thank the reviewers for the positive and constructive comments.

We numbered the comments and provide replies in red.

**the line number mentioned in this reply refers to the MS version with highlighted changes**

1- In addition to the reviewers comments please add CI to the text and figures (to all values of % throughout the manuscript).

When comparing prevalence there should be mention of the p value and CI - only with this information the authors can make statements regarding whether the prevalence reduced or increased - comparing just the % as currently is done is not appropriate.

**We included now in the entire MS the CI and p value, but because of the large sample size in our study we have small confidence intervals and we found statistically significantly differences for measurement that clinically and epidemiologically have in our opinion limited significance. We mention this in the text.**

**For example the prevalence of trichuriasis passed from 82% in 2011 to 75% in 2021, this difference resulted to be significant from the statistical point of view but we did not considered significant from the epidemiological point of view (the same intervention is needed) in particular considering that between the 2 measurement over 15 rounds of deworming were conducted. Therefore despite being statistically significant we consider this difference not relevant.**

**Reviewer #1:**

2-Methods:

The objective of the study is clear. However, since the authors declare that the records of PC from 1994 to 2011 are imprecise it does not seem completely correct to speak about “more than 25 five years of PC”. The statement gives the idea of continuous and consistent PC campaigns during that period which is not the real scenario of the study. Being Pemba a high prevalence area where reinfection is very possible and PC should be implemented twice a year. It is not fear for the PC strategy to evaluate its effectiveness in a setting where: i) the coverage of the PC is imprecise (for example 2016: 47 – 90%) and ii) two years have passed since the last PC round was administered (2019 and 2020).

**We corrected the statement on line 100-101, mentioning that the PC “ intervention has not been consistently administered.”**

3-The design is appropriate for the objective. The diagnostic method used (Kato-Kats) is the recommended by WHO for diagnosis and monitoring of STH. An adequate quality control was also performed.

A definition and description of the formula used to calculate coverage is needed, as well as clarification about the source of the data used for that indicator.

**We included a phrase in line 93-96 explaining the source and the formula used to calculate coverage**

4-More information is needed about population. What ages have the children of grades standard 2 and standard 3?

**We included the corresponding age interval in parenthesis (Line 118-119) and in the results session (line 150-151) the mean age of the two groups**

5-Not all the PC interventions targeted preSAC children. Then children younger than 7 years might have never received PC. The inclusion of those children in the study would underestimate the impact of the PC.

**We add a phrase (line 93) to explain that in the period 2012–2021, reported in the table 1, preschool children, school age children and adults were targeted. All the preschool children (less than 6 years of age and therefore born after 2015 were targeted by the deworming intervention that started, for this age group, in 2012.**

6-The sample size is sufficient; actually the number of children analyzed is impressive.

No ethical concerns arise from reading of the study.

**Thank you**

**Reviewer #2:**

7- One of my main concerns is about the content of methods section. While the methods used were appropriate for this survey, the content of methods section is too brief. More important information should be provided.

For instance, description of the study area, study population, epidemiological characteristics of the targeted population pertaining to intestinal parasites transmission, students’ participation, samples delivery and transport to examination station, etc.

**I addition to the information requested by reviewer#1 point #4 we provided information about:**

- **Time interval in which the survey has been conducted (line 110)**
- **total number of schools in Pemba (line 112)**
- **sex distribution of the sample (line 146)**
- **participation to the survey Line 147**
- **details about transport to examination laboratory line 122-124.**

8- Also, provide information on the selection of schools and the procedures of sampling. It is not enough to just refer the reader to the 2011-study (2018 PhD thesis).

**We included details on the sampling method conducted in 2011 and repeated in 2021 (Line 113)**

9- Please state the time frame of the survey. When was this study conducted?

**The starting and ending dates are now mentioned in line 110**

**Reviewer #1:**

10- The results match the analysis plan and are completely presented.

It would be interesting to know which the hookworm species more prevalent in the island is. Obviously that could not be known from the Kato-Kats results. But maybe that information is known from previous studies or from other laboratory techniques used for control purposes.

**As mentioned by the reviewer this study could not identify hookworm species, however previous studies (Albonico et al 1998) conducted with fecal culture of 524 children both species were found in Pemba: 65% of the children were infected with only *N. americanus*, 2.1% only with *A. duodenale* and 27% with both species (we included this information in line 267).**

11- Figures 4 resume clearly the results. Figures 1, 2 and 3 seem repetitive.

**We agree, we added an additional panel (“any STH”) to figure 4 (now figure 1) and removed figure 1, 2 and 3.**

A map of the island showing the information presented in table 3 would be clarifying and eye-catching.

**We included a map as figure 1**

Reviewer #2: 1.	Line 131: “495 (20%) where not infected,” this can be removed. Please indicate the overall prevalence of STH.

**Done (now line 149-151)**

12 Line 142-143: Add the statistics. Was the difference significant or not? What species? Prevalence of STH by sex: Provide the actual p values where needed. A row can be added to table 2 and p values for all parasite groups can be given.

**The asterisks in table 2 mark where the difference is statistically different by sex and species. Despite being statistically different we consider the difference in prevalence 2011 and 2021 of *A. lumbricoides* and Hookworms irrelevant from the clinical and epidemiological point of view.**

13- Table 3: add rows for total for each district and report statistical tests.

**It was not our intention to evaluate the difference in term of prevalence in the different part of the country but rather to observe the difference in the different years. However, we marked with an asterisk the district that has STH prevalence statistically significantly different from the others.**

14- Table 3: report statistical tests for difference between both regions, i.e. north and south.

**No statistically significant differences were observed between the two regions**

15- Prevalence of STH infection by age: What about difference according to STH species? Please report this and provide statistics.

**No difference between prevalence of the different parasite species in the two age groups considered, we are now mentioning this (Line 166-168)**

16- Prevalence of STH infection by age: this subsection can be merged with the “Prevalence of STH by sex”.

**Done (line 162)**

17-Results according to age can be added to table 2.

**We mentioned in the text (line 170-171) that there is no difference in the prevalence of the different species in the 2 age group**

18- Line 197: “23% in 1994 to 24% in 2021” but this is not a reduction! Please revise these percentages for Ascaris as they are incompatible with Figure 4.

**Text corrected (now line 222)**

18- It will be useful to display the prevalence % over the columns of all figures.

**As requested from reviewer #1 (point #11) we removed figures 1, 2 and “ FIGURE 4 has the % on the Y axis legend**

**Reviewer #1:**

19-The conclusions are supported by the data presented except for the statement: “the interventions did not sufficiently control STH morbidity” (lines 234 and 235). None of the results presented actually measure morbidity (anemia, malnutrition, stunting). Even though it is reasonable to deduce that the morbidity probably remains high with the very high prevalence and infection intensity found.

**The morbidity parameters mentioned by the reviewer (anemia, malnutrition, stunting) are multifactorial and therefore for example in absence of sufficient nutritional intake of iron, anemia can persist in an area where STH have been eliminated.**

**For this reason, since the morbidity caused by STH is proportional to the number of worms infecting the host, WHO recommends to measure STH morbidity evaluating the classes of intensity of worm infection;**

**For example in case of hookworm > than 2000 eggs per gram of faeces correspond to over 5 mg of Hemoglobin lost per g of faeces, a quantity that is difficult to replace in areas of limited iron intake.**

**If no children are in this class of intensity we assume that the hookworm contribution to anemia (hookworm morbidity) has been removed.**

20- The limitations of the analysis are not clearly described. The limitations regarding coverage and records of the PC interventions should be discussed before stating: “In Pemba, the results of a PC intervention conducted for 25 years are not optimal”. Instead it could be more realistic to conclude that: The results of the PC are not optimal with the poor coverage achieved and targeting preSAC in only 4 of the 10 interventions recorded.

**We included a statement on the limitations of the study (line 276-279)**

21- The results show a very interesting difference in response of each STH species to the PC. Trichuris trichiura remains with high prevalence but MHI proportion is reduced. Ascaris lumbricoides shows an important reduction in prevalence but a paradoxical increase in infection intensity. Hookworms show a sustained decrease in prevalence and intensity. These differences should be more fully discussed in the paper for two reasons: 1) they have morbidity implications, and 2) raise the need to establish individual transmission models for each STH species and not seek a single explanation for all. Particularly the findings on hookworms are interesting and require a possible explanation. The efficacy of albendazole is not higher against hookworms than against Ascaris lumbricoides. The reason for the different response of these two species might be an improvement in sanitation facilities for example.

**We thank the reviewer for this suggestion, that we consider extremely important, we provided an explanation to this fact in line 258 -264.**

22- The authors present a possible strategy to improve the results of PC in Pemba based on their results. The option of using mebendazole instead of albendazole should be more fully discussed presenting its advantages and disadvantages.

With this strategy some little efficacy against Trichuris trichiura might be gained but a lot of efficacy against hookworms would be lost.

The strategy of consistently targeting preSAC and adults in the PC interventions should be also discussed.

**We better motivated the suggestion given and the reason why we did not suggest to treat all adults (line 298-302)**

23- The relevance of the study for public health is addressed.

**Thank you**

**Reviewer #2:**

24- Please describe the limitations of the study. For example, using a single faecal sample and single Kato-Katz smear, study design, etc.

**We included a paragraph on the limitation (Line 276-279); however we did not included the performance of the single slide of Kato-Katz as a limitation, since this was done on purpose:**

**In the planning phase of the study we estimated the capacity of our laboratory for this study on approximately 2500 slides, we preferred to examine 1 slide from 2500 children than 2 slides from 1250 children considering that the second slide from the same child normally provide only marginal additional information compared to the first. On the other side a larger sample size allow a much smaller interval of confidence of our results and a better understanding of the epidemiological situation.**

**Reviewer #2:**

25- The abstract in general is suitable for authors summary required by PLoS NTD; and another structured abstract can be provided. In the abstract, results should be provided clearly with proportions and statistics.

**We included statistics in the abstract**

26- Abstract, first sentence: Why only focused on the STH impact on nutritional status? A more general statement can be used.

**We revised the sentence (line 37-38)**

27- Line 39: “periodic treatment” can be replaced by periodic preventive chemotherapy or MDA.

**OK done (line 40)**

28-Lines 41-43: This statement about the 2 surveys can be rewritten in a more general statement to indicate the continued burden over time. The sentence is repeated in the 3rd paragraph of introduction section.

**OK done (line 41-46)**

29- Line 49: “… infections are higher, ..” or remain high!

**OK done (line 53)**

30- Lines 50-52: Did you investigate these factors? This statement is suitable for authors summary but not for the main abstract section.

**Details of the evaluation of these factors are reported in the discussion (line 242-24è)**

31.Lines 57-58: ” points ii & iii” did you investigate these points among the studied population?

**The points indicated by the reviewer were**

**ii) to use mebendazole instead of albendazole because of its better activity on *T. trichiura* and**

**(iii) to use a combination of ivermectin**

**We are intending to implement these measures in the next phase of the control programme**

32- Lines 60-61: Theoretically, Strongyloides stercoralis is also a STH species.

**Of course, however the 3 STH parasites considered in the paper can be diagnosed with the same method and are sensitive to the same donated drugs, for** **this reason are grouped together. Unfortunately for *S. stercoralis* we do not have yet a standardize diagnostic method nor a drug donation.**

33-Table 1: correct “toto” in the title.

**OK done**

34- Table 1: Why PC activities were stopped in 2019 & 2020? Please indicate this in last paragraph of introduction, if applicable.

**OK done line 96-97**

35- Table 1: Correct the “Number of rounds” for lymphatic filariasis in 2018. It cannot be 0!

**Thank you, this was a typo**

36-Line 117: Correct “of ≤ 0.05” to “of < 0.05”.

**Thank you this was a typo**

37- Line 132: remove “only” and “at least”.

**OK done**

38- Line 220: considering point #iii, it is unclear whether the targeted schools and populations are rural or urban.

**We consider the entire island of Pemba as rural area;**

39-The results showed a small increase in the prevalence of MHI in 2021 compared to 2011 (Fig 2) and it seems to be due to Ascaris (Fig 4). This point can be discussed. Was it due to ceased PC activities in 2019-2020?

**This is now discussed line 265 -274**

**Reviewer #1:**

40-This is a very interesting study. Relevant for the area of helminthology and also for public health. It has two major strengths: 1) the long period of follow up and 2) the big sample size. Its weaknesses are: 1) the lack of a global picture since preSAC and adults (especially woman of childbearing age) were not studied and 2) the long period between the last PC intervention and the surveillance study.

**Thank you**

**Reviewer #2:**

41-This manuscript describes a large-scale survey that aimed to determine the prevalence of STH infections among schoolchildren in 4 districts of Pemba Island with the ultimate objective was to assess the impact of more than 25 years of PC on the burden of STH in Pemba. Overall, the study reported beneficial impact of the PC intervention; however, the impact was not optimal due to the lack of other interventions essential to sustain the impact of PC in any endemic area.

**Agreed**

42- Overall, the topic is interesting and important. The findings shed light on the long-term impact of PC intervention in highly endemic areas and hence, worth publication.

**Thank you**

43-However, the manuscript has some drawbacks that should be addressed in a revised version.

**Agreed**

**Reviewer #3:**

44- Shaali Ame and co-authors report the results of a third soil-transmitted helminth (STH) survey among school children in Pemba, this time 25 years after initiation of preventive chemotherapy (PC) in the island. They found a very high Trichuris trichiura prevalence and high Ascaris lumbricoides prevalence while the hookworm prevalence decreased considerably. Various explanations for this persistence of high STH prevalences are discussed.

This is a refreshingly short and clear manuscript, and only few comments are offered for consideration:

**Thank you**

45- Abstract and discussion: while climatic conditions are certainly ideal for STH this probably is not the key factor since they are equally good in many places with lower (albeit often still high) STH prevalences

**In addition to climatic condition we are also citing population density (that is one of the higher in Africa) and lack of sanitation in the abstract line 55-57 and discussion line 244-247.**

46- Abstract and discussion: high T. trichiura transmission is cited as an explanation for the consistently high STH prevalence. However, the transmission obviously is the outcome, not an explanation…

**Agreed, We intended to say that this STH specie that was over 90% prevalent in 1994 is the less sensitive to benzimidazoles. We rephrased the sentences (line 55-56) in the abstract and in the discussion (283-284)**

47- Please state the selection criteria for the surveyed schools in 2011. How representative are they for the island?

**This is now stated (line 111 -121)**

48- Is anything known about treatment coverage in the survey schools and communities? Overall coverage seems to be quite high, though…

Have treatment coverage figures been verified (e.g. with post-treatment surveys)?

**The coverage mentioned in table 1 is the one reported to WHO and verified by WHO**

49- In addition to the infection intensity classes it would be interesting to also add mean/median egg count figures (and compare them with 2011 values, if possible).

**The intensity of infection in epg is now presented in table 2**

50- Not all statistical test results seems to have been reported. E.g. the statistics of between-survey differences are not evident.

**We marked in figure 1 the 2021 prevalence that are statistically different from the ones measured in 2011**

50-No data is presented on STH prevalence among adults.

Most likely, it is also high. In combination with a relatively high proportion of households without or with only unimproved sanitation, they are likely to contribute substantially to transmission. Hence, expanding deworming also to adults might be considered and should at least be discussed among possible options to reduce transmission in the short term.

**We did not collected epidemiological data in adults and we explained the reason why we did not suggest to treat all adults (line 298-302)**

-	Carefully edit the text to eliminate typos, e.g. line 131 “where” should be “were”

**OK the text has been revised by a mother tongue editor**

6Figure Files:

While revising your submission, please upload your figure files to the Preflight Analysis and Conversion Engine (PACE) digital diagnostic tool, <https://pacev2.apexcovantage.com>. PACE helps ensure that figures meet PLOS requirements. To use PACE, you must first register as a user. Then, login and navigate to the UPLOAD tab, where you will find detailed instructions on how to use the tool. If you encounter any issues or have any questions when using PACE, please email us at [figures@plos.org](mailto:figures@plos.org).
